# Early determinants of linear growth and weight attained in the first year of life in a malaria endemic region

**DOI:** 10.1371/journal.pone.0220513

**Published:** 2019-08-06

**Authors:** Juliana Paghi Dal Bom, Lalucha Mazzucchetti, Maíra Barreto Malta, Simone Ladeia-Andrade, Marcia Caldas de Castro, Marly Augusto Cardoso, Bárbara Hatzlhoffer Lourenço, on behalf of the MINA-Brazil Study Working Group

**Affiliations:** 1 Department of Preventive Medicine, Federal University of São Paulo, São Paulo, São Paulo, Brazil; 2 Department of Nutrition, School of Public Health, University of São Paulo, São Paulo, São Paulo, Brazil; 3 Oswaldo Cruz Institute, FIOCRUZ, Rio de Janeiro, Rio de Janeiro, Brazil; 4 Department of Global Health and Population, Harvard T.H. Chan School of Public Health, Boston, Massachusetts, United States of America; Burnet Institute, AUSTRALIA

## Abstract

We investigated linear growth and weight attained among 772 children at 10–15 months of age in the first population-based birth cohort in the Brazilian Amazon. Sociodemographic, maternal and birth characteristics were collected in interviews soon after birth at baseline. Anthropometric evaluation was conducted at 10–15 months. Multiple linear regression models were fitted for length-for-age (LAZ) and body mass index (BMI)-for-age Z scores (BAZ), considering a hierarchical conceptual framework with determinants at distal, intermediate and proximal levels, with adjustment for the child’s sex and age. Mean LAZ and BAZ were 0.31 (SD: 1.13) and 0.35 (SD: 1.06), respectively. Overall, 2.2% of children were stunted and 6.6% overweight. Among socioeconomic factors, household wealth index was positively associated with LAZ (p for trend = 0.01), while children whose families received assistance from the *Bolsa Família* conditional cash transfer program were 0.16 Z score thinner (95% CI: -0.31, -0.00). Maternal height and BMI were positively associated with both LAZ and BAZ at 10–15 months of age (p for trend <0.001). Child’s size at birth was positively related with LAZ (p<0.001 for both birth weight and length). BAZ was 0.34 (95% CI: 0.24, 0.44) higher, but 0.11 lower (95% CI: -0.21, -0.02), for each increase in 1 Z score of birth weight and length, respectively. Children with at least one reported malaria episode within the first year of life were 0.58 (95% CI: -1.05, -0.11) Z score shorter. Socioeconomic and intergenerational factors were consistently associated with LAZ and BAZ at 10–15 months of age. The occurrence of malaria was detrimental to linear growth. In a malaria endemic region, reduction of inequalities and disease burden over the first 1,000 days of life is essential for taking advantage of a critical window of opportunity that can redirect child growth trajectories toward better health and nutrition conditions in the long term.

## Introduction

Assessment of a child’s nutritional status using anthropometric measures allows monitoring the dynamic process of progressive physical changes involving different body compartments, which are essential for proper development [1]. During the last decades, most countries worldwide have experienced significant decreases in the prevalence of length/height-for-age deficits and increases in the prevalence of overweight among children [2,3]. In low- and middle-income countries, such panorama resulted in a double burden of malnutrition affecting 148 million children with stunting (24.3%) and 29 million with overweight (4.8%) up to the age of 5 years in 2018 [4].

Evidence has shown that the first 2 years of life represents a phase of vulnerability that may influence health and human capital in a favorable or adverse fashion [5], depending on exposures during the intrauterine period or soon after birth. Therefore, understanding the factors associated with child growth during this period is relevant to benefit from a window of opportunity to overcome malnutrition and its related effects. In Brazil, recent data from the Pelotas birth cohort study about the time trends of nutritional status have confirmed a 53% decline in the prevalence of stunting and an 88% increase in the prevalence of overweight in children aged 1 year over a 33-year period [6]. Although stunting and overweight were observed in 3.9% and 12.2% of the study participants in 2015, respectively, the study shed light on persistent income-related differences in stunting across decades, which still occur mainly among individuals with the poorest quintile of family income. No association was noted between the prevalence of overweight and socioeconomic status, particularly with the rapid increase in excessive weight among poorer children [6].

Notably, low- and middle-income countries generally present significant social and health inequalities, which results in higher exposure to pathogens, psychosocial stress, micronutrient deficiencies, and inadequate dietary intake, especially among disadvantaged groups, thereby influencing growth during the early years of life [6,7]. Historically, the Brazilian Amazon area has presented with worse indicators of health and nutrition [8], and it is a malaria endemic region, accounting for 98% of malaria cases registered in the country [9]. Using data from mother–child pairs living in the urban area of the city of Cruzeiro do Sul, this study aimed to investigate the predictors of linear growth and weight attained at 10–15 months of age in the first birth cohort study in Western Brazilian Amazon. Identifying potentially modifiable factors associated with growth within the first year of life remains a priority for the timely promotion of adequate conditions among children to achieve their full development potential.

## Materials and methods

### Study design and population

The present study is part of the MINA-Brazil Study (Maternal and Child Health and Nutrition in Acre, Brazil), a population-based birth cohort study conducted in Cruzeiro do Sul, Acre state, Western Brazilian Amazon. The population in the area is approximately 87,673 inhabitants, 70% of whom reside in the urban area. The municipality presented a medium Human Development Index of 0.664 in the 2010 census [10].

The baseline data were obtained between July 1, 2015 and June 30, 2016 in the Women’s and Children’s Hospital of Juruá Valley, where 96% of all deliveries in the municipality take place. Through daily visits by research team members, all women admitted for delivery in the maternity ward were invited to participate after receiving an explanation of the study protocol. Follow-up assessments were scheduled on a weekly basis at a public health center located in the central region of the city, according to the child’s birth date, between July 2016 and June 2017. For each participant, in case of non-attendance, up to three rescheduling attempts were made. Thus, within an age range of 10–15 months, data on maternal and infant characteristics were collected, and anthropometric evaluation was performed.

In the present analyses, singleton live births in the urban area of Cruzeiro do Sul were included. Twins were excluded. Mothers and babies from the remote rural area of the city were not eligible for follow-up. Urban and rural areas were predefined according to contact details collected at baseline regarding the participant’s home address, which was additionally confirmed with the local post office team. Written informed consent was obtained before enrollment. The MINA-Brazil Study was approved by the ethical review board of the School of Public Health, University of São Paulo, Brazil (protocol 872.613, November 13, 2014).

### Data collection and management

All study data were entered in tablets using the Census and Survey Processing System (CSPro, U.S. Census Bureau, ICF International). At baseline, trained research assistants interviewed each mother before discharge from the maternity hospital using a semi-structured questionnaire. Data were collected on maternal age (<19 years, 19–29 years, or ≥30 years), skin color (white, mulatto, and black/yellow/indigenous), educational level (≤9 years, 10–12 years, or >12 years), receiving assistance from the *Bolsa Família* conditional cash transfer program (no or yes), and presence of household assets (television, stereo, computer, DVD player, internet, cable TV, gas stove, refrigerator, blender, electric iron, microwave, sofa set, landline, cell phone, bicycle, motorcycle, car, and land and livestock). Data on household assets were used to calculate wealth index via principal component analysis, which was categorized into quintiles, as a proxy of socioeconomic status for each household [11]. During the interview, information about the total number of antenatal care visits (categorized according to the Brazilian Ministry of Health recommendation in ≤6 or >6 visits [12]) and history of malaria during pregnancy (no or yes) was obtained from the mother’s pregnancy health card. Data were obtained from the hospital records, which included gestational age at delivery (<37 or ≥37 weeks), type of delivery (vaginal or caesarean), child’s sex (female or male), and size at birth. The child’s birth weight and length were measured and registered in the hospital records by obstetric nurses soon after delivery. For analysis, birth weight was categorized into <2,500, 2,500–3,999, or ≥4,000 g, and birth length was classified into tertiles, which were as follows: 1^st^ tertile: ≤48.0 cm, 2^nd^ tertile: 48.1–50.1 cm, and 3^rd^ tertile: >50.1 cm. Regarding gestational age, 34% of the mothers had an ultrasound-confirmed antenatal estimate obtained by our research team, with an acceptable mean difference compared with hospital records (0.43 weeks; 95% confidence interval [CI]: 0.32, 0.53, according to Bland–Altman analysis). Z scores for birth weight and length were also obtained using the INTERGROWTH-21^st^ Project references for gestational age and sex [13].

During follow-up, trained fieldworkers updated the socioeconomic and demographic information and assessed infant feeding practices (including the use of feeding bottles and pacifiers) and occurrence of malaria and pneumonia since birth according to maternal report. Regarding the occurrence of malaria, information about the number of episodes and type of *Plasmodium* was also obtained.

The anthropometric measurements of children and their mothers were obtained in duplicate using standardized procedures and calibrated equipment [14–16]. During measurement, the participants wore light clothes and were barefoot and unadorned with loose hair; the diapers of children were also removed. The child’s recumbent length was measured to the nearest millimeter while the infant lay on a measuring board on flat and firm surface. A portable stadiometer (Alturaexata) with a precision of 0.1 cm and extension of 213 cm was utilized to measure maternal height. Using a digital electronic scale with a capacity of 150 kg (UM061, Tanita Corporation), maternal weight was recorded to the nearest 100 g. Next, the combined weight of the mother and child was obtained while the mother was positioned at the center of the scale, standing upright with feet together, and holding the child in her lap as still as possible. The child’s weight was calculated from the difference between the combined weight measurement and maternal weight. Between duplicates, maximum differences of 0.2 cm and 100 g were accepted for length/height and weight measurements, respectively, and the mean values were calculated. If anthropometric measurements exceeded the maximum allowed differences, a third measurement was obtained, and the mean was calculated based on the two closest values.

Maternal height was categorized into tertiles (1^st^ tertile: ≤155.2 cm, 2^nd^ tertile: 155.3–159.9 cm, and 3^rd^ tertile: ≥160.0 cm). For adult women, maternal body mass index (BMI) was calculated as weight in kilograms divided by height in meters squared. For adolescents, BMI-for-age Z scores (BAZ) were calculated using the World Health Organization (WHO) Anthro Plus software [17]. For the analysis, the maternal BMI of all participants was categorized according to the cut-off points proposed by the WHO into underweight/normal weight, overweight, or obese [15].

Child anthropometric Z scores for length and BMI at 10–15 months of age were calculated according to age and sex using the WHO Child Growth Standards [18]. No implausible Z score values were identified according to the WHO criteria [15]. Stunting was defined as length-for-age Z scores (LAZ) lower than -2.0 Z. Participants with BAZ greater than 2.0 were classified as overweight.

### Data analyses

The outcomes of interest were continuous values of LAZ and BAZ to describe child linear growth and weight attained at 10–15 months of age, respectively. The independent variables included socioeconomic and demographic factors (household wealth index, receiving assistance from the *Bolsa Família* program, maternal education, age, and skin color), maternal characteristics (height, BMI, total number of antenatal care visits, and history of malaria during pregnancy), characteristics at birth (gestational age at delivery, type of delivery, and child’s birth weight and length), and variables related to the child’s first year of life (use of feeding bottle and occurrence of malaria and pneumonia).

Normality assumptions for anthropometric variables were confirmed using the Shapiro–Wilk test. We first compared LAZ and BAZ distributions at 10–15 months of age according to the categories of exposure variables using unpaired t-tests for dichotomous variables and analysis of variance with Bonferroni post-hoc test for ordinal variables. The associations between exposure variables and linear growth and weight attained within the first year of life were analyzed using linear regression models initially adjusted for child’s age and sex (model 1). For each outcome (LAZ and BAZ at 10–15 months), multiple linear regression models were based on a hierarchical conceptual framework [19], considering the associations with exposures at distal (socioeconomic and demographic variables), intermediate (maternal and birth characteristics), and proximal levels (feeding practices and morbidities within the first year of age). At each of these levels of association, variables were retained in the multiple model (model 2) if they were considered conceptually relevant or if significantly associated with the outcome (p <0.05). Thus, in model 2, regression coefficients were estimated after adjusting for child’s age, sex, and exposures retained in the preceding levels of association. To assess for potential collinearity, variance inflation factors (VIF) of model 2 for both LAZ and BAZ were assessed. The mean VIFs were 1.23 and 1.25, respectively, without indication for multicollinearity among predictors.

For all models, we calculated regression coefficients and their 95% CI among the categories of each exposure. All analyses were performed using Stata 14.0 (StataCorp, College Station, TX, USA).

## Results

**Fig 1:**
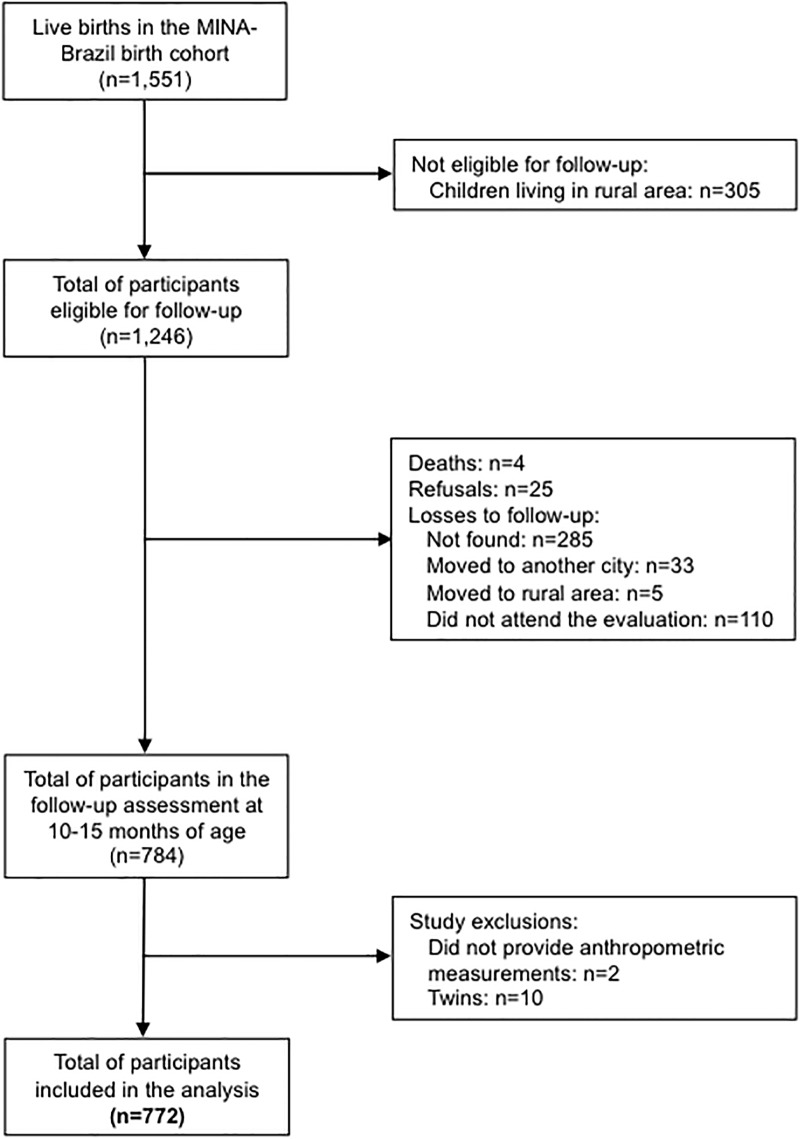
Flowchart of participants in the MINA-Brazil Study for analysis of linear growth and weight attained at 10–15 months of age, Cruzeiro do Sul, Acre, Brazil.

From 1,551 live births enrolled in the MINA-Brazil Study, 1,246 children were considered eligible for follow-up, of whom 305 were living in remote rural areas. During follow-up, there were 4 deaths and 25 refusals, and 433 participants were lost to follow-up. Two children did not provide anthropometric measurements during follow-up and 10 were twin births. Thus, during the assessment at 10–15 months (mean age: 12.7 months; standard deviation [SD]: 0.73), 772 children had complete anthropometric information (62.0% of those eligible) (Fig 1), of whom 52.2% were girls. The mean LAZ was 0.37 (SD: 1.13) for girls and 0.26 (SD: 1.13) for boys. Of these, 2.0% of girls and 2.4% of boys were stunted. The mean BAZ of girls was 0.41 (SD: 1.06) and that of boys was 0.38 (SD: 1.07). Of these, 6.7% of girls and 6.0% of boys were overweight. No significant differences were observed in terms of sex.

Distributions of LAZ and BAZ at 10–15 months of age by of socioeconomic, maternal, and child characteristics are presented in Table 1. At baseline, 39.3% of households received assistance from the *Bolsa Família* program, and 29.8% of the mothers had up to 9 years of education. Overall, 16.1% of the mothers were teenagers, 30.5% did not receive the minimum recommended number of six antenatal care visits, and 6.5% had malaria during pregnancy. At birth, the mean weight and length for gestational age Z scores were 0.19 (SD: 0.99) and 0.11 (SD: 1.05), respectively, according to the references from the INTERGROWTH-21^st^ Project. Regarding the occurrence of disease within the first year of life, 2.7% of the children were affected by malaria. Among them, 46.6% had more than one episode within the first year of life. Among the 21 reported malaria episodes, there were 14 cases of *Plasmodium vivax* infections; 4 cases of *P*. *falciparum* infection; and 1 case of mixed infection. For two cases, the parasite species was unknown. Remarkably, occurrence of malaria during the first year of life was more frequent among children whose mothers presented with malaria during pregnancy (14.0% versus 1.9% of children whose mothers did not present with malaria during pregnancy, Fisher exact test: p <0.001). Pneumonia was reported for 3.4% of children.

**Table 1:** Mean length-for-age Z score (LAZ) and BMI-for-age Z score (BAZ) at 10–15 months of age according to socioeconomic, maternal and child characteristics, MINA-Brazil Study, Cruzeiro do Sul, Acre, Brazil.

Variables	N (%)	Mean LAZ (SD)^a^^,^^c^	P	Mean BAZ (SD) ^b^^,^^c^	P
**Child’s sex (n = 772)**			0.18		0.76
Female	403 (52.2)	0.37 (1.13)		0.41 (1.06)	
Male	369 (47.8)	0.26 (1.13)		0.38 (1.07)	
**Household wealth index (n = 760)**					
1^st^ quintile	123 (16.2)	0.09 (1.11)	Ref.	0.38 (0.86)	Ref.
2^nd^ quintile	148 (19.5)	0.31 (1.20)	1.00	0.42 (1.09)	1.00
3^rd^ quintile	167 (22.0)	0.35 (1.13)	0.46	0.40 (1.09)	1.00
4^th^ quintile	150 (19.7)	0.37 (1.08)	0.41	0.37 (1.13)	1.00
5^th^ quintile	172 (22.6)	0.48 (1.09)	0.03	0.39 (1.09)	1.00
**Assistance from the *Bolsa Família* program (n = 760)**			0.34		0.08
No	479 (63.0)	0.36 (1.16)		0.44 (1.07)	
Yes	281 (37.0)	0.28 (1.08)		0.31 (1.05)	
**Maternal education (years, n = 759)**					
≤9	226 (29.8)	0.17 (1.13)	Ref.	0.32 (1.02)	Ref.
10–12	321 (42.3)	0.38 (1.14)	0.11	0.41 (1.08)	0.96
>12	212 (27.9)	0.44 (1.09)	0.03	0.43 (1.09)	0.83
**Maternal age (years, n = 772)**					
<19	124 (16.1)	0.19 (1.18)	Ref.	0.54 (1.02)	Ref.
19–29	423 (54.7)	0.39 (1.13)	0.24	0.42 (1.06)	0.80
≥30	225 (29.2)	0.27 (1.10)	1.00	0.26 (1.09)	0.06
**Maternal skin color (n = 760)**					
White	93 (12.2)	0.33 (0.99)	Ref.	0.20 (1.00)	Ref.
Mulatto	598 (78.7)	0.32 (1.13)	1.00	0.44 (1.04)	0.15
Black, yellow, indigenous	69 (9.1)	0.42 (1.30)	1.00	0.28 (1.30)	1.00
**Total number of antenatal care visits (n = 768)**			0.05		0.11
≤6	234 (30.5)	0.20 (1.22)		0.30 (1.03)	
>6	534 (69.5)	0.37 (1.08)		0.43 (1.08)	
**Maternal height (cm, n = 755)**					
1^st^ tertile: ≤155.2	253 (33.4)	0.00 (1.13)	Ref.	0.45 (0.97)	Ref.
2^nd^ tertile: 155.3–159.9	251 (33.3)	0.20 (1.08)	0.12	0.23 (1.03)	0.05
3^rd^ tertile: ≥160.0	251 (33.3)	0.75 (1.06)	<0.001	0.45 (1.14)	1.00
**Maternal nutritional status (n = 750)**					
Underweight / Normal weight	414 (55.2)	0.31 (1.14)	Ref.	0.31 (1.03)	Ref.
Overweight	229 (30.5)	0.30 (1.12)	1.00	0.39 (1.07)	0.97
Obesity	107 (14.3)	0.38 (1.17)	1.00	0.61 (1.08)	0.02
**History of malaria during pregnancy (n = 772)**			0.60		0.85
No	722 (93.5)	0.32 (1.13)		0.39 (1.08)	
Yes	50 (6.5)	0.40 (1.16)		0.42 (0.86)	
**Gestational age at delivery (weeks, n = 772)**			<0.001		0.52
<37	60 (7.8)	-0.25 (1.24)		0.31 (0.90)	
≥37	712 (92.2)	0.37 (1.11)		0.40 (1.08)	
**Type of delivery (n = 772)**			0.13		0.02
Vaginal	400 (51.8)	0.26 (1.07)		0.31 (1.05)	
Caesarean	372 (48.2)	0.39 (1.19)		0.48 (1.07)	
**Birth weight (g, n = 771)**					
<2,500	49 (6.4)	-0.56 (1.20)	Ref.	0.09 (0.88)	Ref.
2,500–3,999	675 (87.6)	0.35 (1.10)	<0.001	0.38 (1.07)	0.18
≥4,000	47 (6.1)	0.79 (1.09)	<0.001	0.86 (0.98)	<0.001
**Birth length (cm, n = 768)**					
1^st^ tertile: ≤48.0	252 (32.8)	-0.07 (1.13)	Ref.	0.23 (1.06)	Ref.
2^nd^ tertile: 48.1–50.1	330 (43.0)	0.36 (1.06)	<0.001	0.47 (1.06)	0.02
3^rd^ tertile: >50.1	186 (24.2)	0.80 (1.04)	<0.001	0.48 (1.05)	0.05
**Use of feeding bottle at 12 months of age (n = 772)**			0.02		0.71
No	288 (37.3)	0.20 (1.08)		0.41 (1.09)	
Yes	484 (62.7)	0.40 (1.16)		0.38 (1.05)	
**Malaria within the first year of life (n = 772)**			0.02		0.45
No	751 (97.3)	0.34 (1.13)		0.40 (1.07)	
Yes	21 (2.7)	-0.24 (1.16)		0.22 (0.99)	
**Pneumonia within the first year of life (n = 772)**			0.01		0.43
No	746 (96.6)	0.34 (1.13)		0.39 (1.07)	
Yes	26 (3.4)	-0.21 (1.09)		0.55 (0.85)	

^a^LAZ: length-for-age Z score, calculated according to the WHO growth standards (WHO, 2006).

^b^BAZ: BMI-for-age Z score, calculated according to the WHO growth standards (WHO, 2006).

^c^Comparison of anthropometric indices according to the categories of exposure variables using unpaired t-tests for dichotomous variables and analysis of variance with Bonferroni post-hoc test for ordinal variables.

Table 2 shows the factors associated with linear growth at 10–15 months of age. Considering adjusting for child’s age and sex (model 1), wealth index, maternal education, total number of antenatal care visits, type of delivery, birth weight and length Z scores, and use of feeding bottle were positively associated with LAZ, whereas the occurrence of malaria and pneumonia within the first year of life had a negative impact on linear growth. After multiple adjustment (model 2), the household wealth index at baseline was found to be positively associated with LAZ at 10–15 months (p for trend = 0.01), with a difference of 0.39 Z score (95% CI: 0.13, 0.65) in children in the richest quintile of wealth compared to those in the poorest quintile. After additional adjustment for wealth, maternal height was still positively associated with LAZ across tertiles (p for trend <0.001). Similarly, although attenuation of associations was noted with further adjustment for wealth and maternal height, birth weight and length Z scores remained positively associated with linear growth within the first year of life. An increase of 1 Z score of birth weight and length for gestational age corresponded to a mean LAZ 0.17 (95% CI: 0.07, 0.27) and 0.15 (95% CI: 0.05, 0.25) higher within the first year (p <0.001), respectively. The analysis with categories of birth weight and length had similar findings. Of note, the occurrence of malaria negatively impacted linear growth at 10–15 months even after adjusting for socioeconomic, maternal, and birth characteristics. Children with at least one reported malaria episode within the first year of life had a Z score 0.58 (95% CI: -1.05, -0.11) lower than those without reported episodes. Among children affected by malaria, two were stunted at 10–15 months of age (9.5% versus 2.0% of children who were not affected by malaria, Fisher exact test: p = 0.08).

**Table 2:** Adjusted linear regression coefficients of factors associated with length-for-age Z score (LAZ) at 10–15 months of age, MINA-Brazil Study, Cruzeiro do Sul, Acre, Brazil.

Variables	Model 1^a^		Model 2^b^	
	ß	95%CI	ß	95%CI
**Distal factors**				
**Household wealth index (n = 760)**				
1^st^ quintile	Ref.		Ref.	
2^nd^ quintile	0.22	-0.05, 0.49	0.22	-0.05, 0.49
3^rd^ quintile	0.27	0.01, 0.53	0.27	0.01, 0.53
4^th^ quintile	0.28	0.01, 0.54	0.28	0.01, 0.54
5^th^ quintile	0.39	0.13, 0.65	0.39	0.13, 0.65
**Assistance from the *Bolsa Família* program (n = 760)**	-0.08	-0.25, 0.08		
**Maternal education (years, n = 759)**				
<9	Ref.			
10–12	0.22	0.02, 0.41		
>12	0.28	0.07, 0.49		
**Maternal age (years, n = 772)**				
<19	Ref.			
19–29	0.21	-0.02, 0.43		
≥30	0.08	-0.17, 0.32		
**Maternal skin color (n = 760)**				
White	Ref.			
Mulatto	-0.01	-0.26, 0.24		
Black, yellow, indigenous	0.08	-0.27, 0.43		
**Intermediate factors**				
**Total number of prenatal care visits (n = 768)**				
≤6	Ref.			
>6	0.17	-0.00, 0.35		
**Maternal height (cm, n = 751)**				
1^st^ tertile: ≤155.2	Ref.		Ref.	
2^nd^ tertile: 155.3–159.9	0.20	0.01, 0.39	0.21	0.01, 0.40
3^rd^ tertile: ≥160.0	0.75	0.55, 0.94	0.72	0.52, 0.91
**Maternal nutritional status (n = 750)**				
Underweight / Normal weight	Ref.			
Overweight	-0.01	-0.20, 0.17		
Obesity	0.06	-0.18, 0.30		
**History of malaria during pregnancy (n = 772)**	0.08	-0.24, 0.41		
**Proximal factors**				
**Gestational age at delivery (weeks, n = 772)**				
<37	Ref.			
≥37	0.63	0.33, 0.92		
**Type of delivery (n = 772)**				
Vaginal	Ref.			
Caesarean	0.07	-0.01, 0.15		
**Birth weight Z-score (n = 771)**	0.31	0.23, 0.39	0.17	0.07, 0.27
**Birth length Z-score (n = 768)**	0.29	0.22, 0.36	0.15	0.05, 0.25
**Use of feeding bottle at 12 months of age (n = 772)**	0.20	0.03, 0.36		
**Malaria within the first year of life (n = 772)**	-0.58	-1.07, -0.09	-0.58	-1.05, -0.11
**Pneumonia within the first year of life (n = 772)**	-0.54	-0.99, -0.10	-0.41	-0.85, 0.04

^a^Model 1: linear regression models with adjustment for child’s age and sex.

^b^Model 2: multiple linear regression models with adjustment by child’s age and sex and exposures retained in the preceding levels of association, according to a hierarchical conceptual framework for factors associated with LAZ.

Table 3 shows the factors associated with weight attained at 10–15 months of age. After adjusting for child’s age and sex (model 1), total number of prenatal care visits, maternal skin color, maternal BMI, type of delivery, and birth weight and length Z scores were found to be positively associated with BAZ. Receiving assistance from the *Bolsa Família* program and maternal age were inversely associated with the outcome. In multiple regression models (model 2), children whose families received assistance from the *Bolsa Família* program presented a mean BAZ 0.16 lower during the first year of life (95% CI: -0.31, -0.00). The Z score of children whose mothers were aged ≥30 years was 0.25 lower than that of children with adolescent mothers (p for trend = 0.04). After adjusting for socioeconomic and demographic variables, maternal BMI was still positively associated with the child’s BAZ at 10–15 months of age (p for trend <0.001). After further adjusting for variables in distal and intermediate levels of association, an increase of 1 Z score of birth weight implied a mean BAZ 0.34 (95% CI: 0.24, 0.44) higher within the first year of life. Conversely, birth length Z score was negatively associated with BAZ at 10–15 months of age (-0.11; 95% CI: -0.21, -0.02). The associations still had the same direction and similar magnitude when considering an alternative analysis with categories of birth weight and length.

**Table 3:** Adjusted linear regression coefficients of factors associated with BMI-for-age Z score (BAZ) at 10–15 months of age, MINA-Brazil Study, Cruzeiro do Sul, Acre, Brazil.

Variables	Model 1^a^		Model 2^b^	
	ß	95%CI	ß	95%CI
**Distal factors**				
**Household wealth index (n = 760)**				
1^st^ quintile	Ref.			
2^nd^ quintile	0.02	-0.23, 0.28		
3^rd^ quintile	0.02	-0.23, 0.27		
4^th^ quintile	-0.02	-0.27, 0.24		
5^th^ quintile	0.00	-0.24, 0.25		
**Assistance from the *Bolsa Família* program (n = 760)**	-0.14	-0.30, 0.01	-0.16	-0.31, -0.00
**Maternal education (years, n = 742)**				
≤9	Ref.			
10–12	0.09	-0.09, 0.27		
>12	0.11	-0.09, 0.31		
**Maternal age (years, n = 772)**				
<19	Ref.		Ref.	
19–29	-0.13	-0.35, 0.08	-0.17	-0.39, 0.04
≥30	-0.28	-0.52, -0.05	-0.25	-0.49, -0.02
**Maternal skin color (n = 760)**				
White	Ref.		Ref.	
Mulatto	0.23	0.00, 0.47	0.23	-0.01, 0.46
Black, yellow, indigenous	0.08	-0.25, 0.41	0.09	-0.24, 0.42
**Intermediate factors**				
**Total number of prenatal care visits (n = 768)**				
≤6	Ref.			
>6	0.13	-0.03, 0.29		
**Maternal height (cm, n = 751)**				
1^st^ tertile: ≤155.2	Ref.			
2^nd^ tertile: 155.3–159.9	-0.22	-0.41, -0.04		
3^rd^ tertile: ≥160.0	-0.00	-0.19, 0.18		
**Maternal nutritional status (n = 750)**				
Underweight / Normal weight	Ref.		Ref.	
Overweight	0.08	-0.09, 0.25	0.17	-0.01, 0.34
Obesity	0.32	0.09, 0.54	0.43	0.21, 0.66
**History of malaria during pregnancy (n = 772)**	0.04	-0.26, 0.35		
**Proximal factors**				
**Gestational age at delivery (weeks, n = 771)**				
<37	Ref.			
≥37	0.09	-0.19, 0.37		
**Type of delivery (n = 772)**				
Vaginal	Ref.			
Caesarean	0.08	0.01, 0.16		
**Birth weight Z-score (n = 771)**	0.26	0.18, 0.33	0.34	0.24, 0.44
**Birth length Z-score (n = 768)**	0.11	0.04, 0.18	-0.11	-0.21, -0.02
**Use of feeding bottle at 12 months of age (n = 772)**	-0.02	0.18, 0.13		
**Malaria within the first year of life (n = 772)**	-0.16	-0.62, 0.30		
**Pneumonia within the first year of life (n = 772)**	0.16	-0.26, 0.58		

^a^Model 1: linear regression models with adjustment for child’s age and sex.

^b^Model 2: multiple linear regression models with adjustment by child’s age and sex and exposures retained in the preceding levels of association, according to a hierarchical conceptual framework for factors associated with BAZ.

## Discussion

Based on data from the MINA-Brazil Study, on average, linear growth and weight attained at 10–15 months of age were 0.32 and 0.39 Z scores, respectively, according to the WHO growth standards. In addition, the frequency of stunting (2.2%) was lower than that of overweight (6.6%) within the first year of life, and no differences were observed in terms of sex. Socioeconomic, maternal, and birth characteristics were associated with these outcomes. In addition, the occurrence of malaria during the first year of life had a significant negative impact on LAZ at 10–15 months of age, and no association was noted between the occurrence of malaria and BAZ.

Almost all children with stunting are born in low- and middle-income regions. However, the prevalence of stunted growth decreased by 25.3% and 65.7% in middle-low and middle-high income countries, respectively, among children aged up to 5 years between 2000 and 2018. Regarding overweight during the same period, the prevalence increased by 30.1% and 10.9% in this age group for these countries, respectively [4]. Our findings were in accordance with these temporal trends within the child’s first year of life.

The household wealth index was associated with child linear growth at this early stage of life. Data from 79 low- and middle-income countries have indicated that the prevalence of stunting among children up to 5 years was 2.5 times higher among those from the poorest quintile of wealth than in those from the richest quintile [3], and this phenomenon has been recently observed in 12-month-old children in Brazil in a time trend study using data from the Pelotas birth cohort study between 1982 and 2015 [6]. Considering LAZ as a continuous variable, similarly to the present analysis, a previous population-based longitudinal study in the Brazilian Amazon has shown that socioeconomic context (measured through household wealth and land ownership) was an important determinant of linear growth in school-aged children, particularly after 2 years of age [20].

By contrast, as reported at baseline, receiving assistance from the *Bolsa Família* program was associated with a slightly lower BAZ within the first year of life in the present study. No significant relationship was observed between receiving assistance from the *Bolsa Família* program and LAZ. Conceived as a strategy to reduce inequality and overcome poverty, effects of the *Bolsa Família* program for better nutritional outcomes were observed after the second year of life, considering the categorical anthropometric indicators of stunting and underweight in a cross-sectional analysis with 22,375 impoverished children under 5 years of age [21]. A population-based longitudinal analysis of children born in 2004 in the South region of Brazil has shown an inverse association between receiving assistance from the *Bolsa Família* program since birth and Z scores of weight and length achieved at 12 months of age [22]. These findings were of similar magnitude to those observed in our analysis. As heterogeneity among sites exposed to conditional cash transfer programs must be recognized, caution should be observed when interpreting the negative association between receiving assistance from the *Bolsa Família* program and BAZ. First, the mean difference of -0.16 for BAZ among children from families receiving assistance from the program was observed after a relatively short follow-up (12 months). Also, from 2000 to 2010 [10], a period in which the *Bolsa Família* program has been expanded nationwide in Brazil, our study area has evolved overall from low to intermediate HDI level. Although critical gaps in social and health conditions in areas such as the Brazilian Amazon are persistent, we observed that approximately 10% of children in our study population had BAZ values below -1, and, conversely, one-fourth of children had BAZ values above +1. This is of special concern considering that most health and nutrition policies have not been adapted to the current epidemiological scenario and still focus on decreasing the incidence of child undernutrition [23].

Consistent positive associations were observed between maternal nutritional status and both LAZ and BAZ at 10–15 months of age in the present study. Data from five cohort studies conducted in low- and middle-income countries have shown the same association at 2 years of age, with a 0.08 higher LAZ achieved with every 1 cm increase in maternal height (95% CI: 0.08, 0.09) [24]. Cross-sectional data of 3,676 children under the age of 5 years based on the latest National Demographic and Health Survey in Brazil have indicated a positive association between maternal BMI and child BAZ (β = 0.17, p <0.001) [25], which was also observed in a longitudinal analysis conducted in the Brazilian Amazon—children whose mothers were overweight or obese had a mean BAZ 0.73 higher (95% CI: 0.11; 1.35) at the age of 12 months [26]. Along with socioeconomic factors, notably in low- and middle-income countries marked by cycles of inequality, intergenerational influences (as represented by maternal nutritional status) have a structural role in shaping the potential growth of children within the first 1,000 days.

In the current study, child’s birth size was associated with linear growth and weight attained at 10–15 months of age after adjusting for socioeconomic and maternal characteristics, which is supported by previous evidence [20,26]. Independently of each other, both birth weight and length were positively associated with LAZ within the first year of life. Moreover, a positive association was observed between BAZ at 10–15 months and birth weight, but birth length was inversely related to weight attained within the first year of life in the final model. Fetal growth is an important predictor of growth patterns in childhood; thus, the joint study of newborn size and growth in the early years of life is important to understand the risks of and improve the prognosis of diseases in later life [27]. Considering weight and length at birth as summary measures of growth during the intrauterine period, our findings suggest that these concurrent growth dimensions had contrasting influences on BAZ attained at a very early stage of life, which in turn may be regarded as a proxy of subsequent adiposity. Interestingly, in the Generation R Study, femur length during the second trimester according to ultrasound examinations was inversely correlated to child BMI at the adiposity peak, which occurred at around 9 months of age [28]. In fact, prospective evidence has shown that such pattern of association seems to track over the years. A report from the 1993 Pelotas birth cohort study has indicated that conditional length gains at 0–6 and 6–12 months of age, after adjusting for conditional weight accretions in each of these periods, were negatively associated with BMI and triceps and subscapular skin folds during adolescence at the age of 14–15 years [29]. Analogous results were observed during a longer follow-up period in the MRC National Survey of Health and Development. Increases in height from 2 to 20 years of age after adjusting for weight gains at the same intervals were associated with lower fat/lean mass ratio, which was measured using a DXA scanner at 60–64 years of age [30]. It is possible that favoring linear growth in relation to simultaneous weight gain may result in a positive body composition profile, with likely repercussion on the risk of chronic disease afterward.

Although our data have not indicated a substantial nutritional risk for stunting in view of the prevalence of length-for-age Z scores below -2, the present study identified that the occurrence of malaria during the first year of life was negatively associated with linear growth at 10–15 months of age after adjusting for socioeconomic, maternal, and birth characteristics. No association was observed between the occurrence of malaria during the first year of life and BAZ. In a recent literature review [31], longitudinal data mainly from African countries were not sufficient to characterize a causal association between malaria and stunting among children. However, authors have emphasized that the studies focused on cases of *P*. *falciparum* infections. The findings about *P*. *vivax* infection, which is predominant in Amazonian regions, may differ due to the presence of hypnozoites, a dormant form of the parasite associated with relapses and chronicity of the disease [31]. In the Peruvian Amazon, longitudinal data of 442 children aged 0–72 months have indicated a growth velocity 0.07 cm slower per malaria episode as diagnosed using thick blood smear after 4 months of follow-up (95% CI: -0.137, -0.004) and 0.08 cm slower per episode after 6 months (95% CI: -0.151, -0.015) [32]. In the Brazilian Amazon, prospective associations between malaria and linear growth in children <5 years have not been confirmed to date due to the limited number of participants in these younger age groups [33].

Recently, the relationship between malaria episodes during pregnancy, as retrieved from the electronic malaria notification system of the Brazilian Ministry of Health, and child’s birth size was analyzed among 1,180 women. Birth length was 0.31 Z score (95% CI: 0.08; 0.54) or 0.47 cm (95% CI: 0.05, 0.88) shorter in children whose mothers presented with malaria during pregnancy than in those whose mothers did not present with such disease [34]. According to a prospective analysis of 303 mother–child pairs in Burkina Faso, antenatal malaria exposure may affect the development of fetal and infant innate immunity through lower levels of cytokines, chemokines, and growth factors, which resulted in a higher risk for malaria infection from age 6 to 12 months [35]. In addition, in Kenya, longitudinal data have shown that, although children in high-transmission regions of malaria had higher levels of maternal antibodies and immunity against infections during the first 6 months of life, the antibody levels decreased and a higher incidence of malaria caused by *P*. *falciparum* was observed in these children after this period [36]. An environment with increased disease burden, which often exposes children to infection since conception, was more likely to increase the risk of disease particularly after 6 months after birth, which may occur in an analogous way in malaria endemic areas in the Brazilian Amazon. In the present analysis, the occurrence of malaria during the first year of life was significantly more common in children whose mothers had a history of malaria during pregnancy than in those with mothers without any history. Finally, the present study showed the negative impact of malaria on child linear growth within the first year of life, and such condition is a potentially modifiable factor. Such understanding has the greatest weight considering that after decreases in the number of malaria cases for several years, a significant increase in such disease in Cruzeiro do Sul was observed during the data collection of the MINA-Brazil Study [9].

The present study had some limitations and strengths. Although this was a population-based study, some participants were lost to follow-up, which was more common among children born to younger and poorer mothers. In addition, child weight was not directly measured and was obtained from the difference between the combined weight of mother and child and maternal weight. However, this approach is suggested in the WHO child growth assessment training materials for children under 2 years of age [16]. Our research team gathered most of the data from hospital records and health cards, which are the common sources of information in the official health registries. However, the occurrence of malaria and pneumonia during the first year of life were confirmed based on maternal report. Even though this is a relatively short period of time, recall bias cannot be ruled out. The present analysis also lacked biochemical indicators from mothers and their children that could have an impact on linear growth and weight attained at 10–15 months of age. Regarding follow-up, it is important to point out that growth faltering usually manifests most incisively at around 2 years, which indicates that nutritional deficits may worsen after 10–15 months of age [37,38], and our analysis did not cover this period. Nonetheless, our results were based on data collected prospectively in an understudied area, with continuous mechanisms for consistency checking, and several factors associated with child growth at an early age were assessed, in order to inform potential interventions.

## Conclusion

The mean LAZ and BAZ within the first year of life in the MINA-Brazil Study were similar to the WHO growth standards and the occurrence of overweight was higher than that of stunting. Socioeconomic factors, maternal nutritional status, and child size at birth were associated with LAZ and BAZ at 10–15 months of age. The occurrence of malaria within the first year of life was significantly related to worse LAZ. Focusing on an early stage of childhood, the present study emphasized the importance of directing actions to reduce inequalities and improve health conditions when facing a double burden of malnutrition, taking advantage of a window of opportunity that may redirect child growth trajectories toward better short- and long-term health and nutrition outcomes.
